# The creation of the Global Scales for Early Development (GSED) for children aged 0–3 years: combining subject matter expert judgements with big data

**DOI:** 10.1136/bmjgh-2022-009827

**Published:** 2023-01-17

**Authors:** Gareth McCray, Dana McCoy, Patricia Kariger, Magdalena Janus, Maureen M Black, Susan M Chang, Fahmida Tofail, Iris Eekhout, Marcus Waldman, Stef van Buuren, Rasheda Khanam, Sunil Sazawal, Ambreen Nizar, Yvonne Schönbeck, Arsène Zongo, Alexandra Brentani, Yunting Zhang, Tarun Dua, Vanessa Cavallera, Abbie Raikes, Ann M Weber, Kieran Bromley, Abdullah Baqui, Arunangshu Dutta, Imran Nisar, Symone B Detmar, Romuald Anago, Pacifico Mercadante, Fan Jiang, Raghbir Kaur, Katelyn Hepworth, Marta Rubio-Codina, Samuel N Kembou, Salahuddin Ahmed, Gill A Lancaster, Melissa Gladstone

**Affiliations:** 1 School of Medicine, Keele University, Keele, UK; 2 Harvard Graduate School of Education, Cambridge, Massachusetts, USA; 3 Consultant, WHO, Geneve, Switzerland; 4 Offord Centre for Child Studies, Department of Psychiatry and Behavioural Neurosciences, McMaster University, Hamilton, Ontario, Canada; 5 Department of Pediatrics, University of Maryland School of Medicine, Baltimore, Maryland, USA; 6 RTI International, Research Triangle Park, North Carolina, USA; 7 Caribbean Institute for Health Research, The University of the West Indies, Kingston, Jamaica; 8 Nutrition and Clinical Services Division, International Centre for Diarrhoeal Disease Research Bangladesh, Dhaka, Bangladesh; 9 Department of Child Health, TNO, Leiden, The Netherlands; 10 College of Public Health, University of Nebraska Medical Center, Omaha, Nebraska, USA; 11 Department of International Health, Johns Hopkins, Baltimore, Maryland, USA; 12 Center for Public Health Kinetics, New Delhi, New Delhi, India; 13 Department of Pediatrics and Child Health, Faculty of Health Sciences, Medical College, The Aga Khan University, Karachi, Pakistan; 14 Innovations for Poverty Action, Washington, District of Columbia, USA; 15 Pediatrics, Universidade de Sao Paulo Faculdade de Medicina, Sao Paulo, Brazil; 16 Shanghai Children's Medical Center Affiliated to Shanghai Jiaotong University School of Medicine, Shanghai, Shanghai, China; 17 National Children's Medical Center, Shanghai Children's Medical Center affiliated to School of Medicine, Shanghai Jiao Tong University, Shanghai, China; 18 Brain Health Unit, Mental Health and Substance Use Department, World Health Organization, Geneve, Switzerland; 19 School of Public Health, University of Nevada Reno, Reno, Nevada, USA; 20 International Center for Maternal and Newborn Health, Johns Hopkins University Bloomberg School of Public Health, Baltimore, Maryland, USA; 21 Projahnmo Research Foundation, Dhaka, Bangladesh; 22 Paediatrics, Aga Khan University, Karrachi, Pakistan; 23 University of Nebraska-Lincoln College of Education and Human Sciences, Lincoln, Nebraska, USA; 24 Inter-American Development Bank, Washington, District of Columbia, USA; 25 Women and Children's Health, University of Liverpool, Liverpool, UK

**Keywords:** Child health, Other study design, Paediatrics

## Abstract

**Introduction:**

With the ratification of the Sustainable Development Goals, there is an increased emphasis on early childhood development (ECD) and well-being. The WHO led Global Scales for Early Development (GSED) project aims to provide population and programmatic level measures of ECD for 0–3 years that are valid, reliable and have psychometrically stable performance across geographical, cultural and language contexts. This paper reports on the creation of two measures: (1) the GSED Short Form (GSED-SF)—a caregiver reported measure for population-evaluation—self-administered with no training required and (2) the GSED Long Form (GSED-LF)—a directly administered/observed measure for programmatic evaluation—administered by a trained professional.

**Methods:**

We selected 807 psychometrically best-performing items using a Rasch measurement model from an ECD measurement databank which comprised 66 075 children assessed on 2211 items from 18 ECD measures in 32 countries. From 766 of these items, in-depth subject matter expert judgements were gathered to inform final item selection. Specifically collected were data on (1) conceptual matches between pairs of items originating from different measures, (2) developmental domain(s) measured by each item and (3) perceptions of feasibility of administration of each item in diverse contexts. Prototypes were finalised through a combination of psychometric performance evaluation and expert consensus to optimally identify items.

**Results:**

We created the GSED-SF (139 items) and GSED-LF (157 items) for tablet-based and paper-based assessments, with an optimal set of items that fit the Rasch model, met subject matter expert criteria, avoided conceptual overlap, covered multiple domains of child development and were feasible to implement across diverse settings.

**Conclusions:**

State-of-the-art quantitative and qualitative procedures were used to select of theoretically relevant and globally feasible items representing child development for children aged 0–3 years. GSED-SF and GSED-LF will be piloted and validated in children across diverse cultural, demographic, social and language contexts for global use.

**WHAT IS ALREADY KNOWN ON THIS TOPIC**

- Accurate measurement of early childhood development (ECD) that is comparable across countries is essential to monitor whether (1) countries are meeting developmental targets and (2) child development intervention programmes have successfully impacted children’s development.

**WHAT THIS STUDY ADDS**

- We created two measures, the Global Scales of Early Development, sharing a common scale that measure ECD at the population and programmatic levels. The measures, for children aged 0–3 years, include items with adequate psychometric properties derived from 18 instruments used across 32 countries, which were curated through consensus by subject matter experts.

**HOW THIS STUDY MIGHT AFFECT RESEARCH, PRACTICE OR POLICY**

- Programme managers and policy makers will have a set of open-access ECD measures with excellent psychometric properties that can be used across programmes and populations globally, in a comparable manner, with minimal training and implementation burden.

## Introduction

With the ratification of the Sustainable Development Goals (SDGs), there has been an increased focus on early childhood development (ECD)^1^ with the need for countries to estimate the rates of children ‘developmentally on track’, as required by SDG target 4.2.1. Until recently, the development of children under 3 years in global contexts has usually been estimated through the use of proxy measures such as stunting and poverty^2^ due to the lack of validated instruments for measuring development rigorously, feasibly and equitably for this age group across national, cultural, demographic, social and language contexts.^1^ Ongoing issues include the reliability and validity of measuring child development across contexts, the cultural sensitivity of specific items,^2^ and the appropriateness of developmental instruments created in Western Educated, Industrial, Rich and Democratic (WEIRD) contexts for use globally.^3 4^ To address these issues, several culturally appropriate instruments have been developed for use in low resource, non-WEIRD settings, including the Malawi Developmental Assessment Tool,^5^ the Kilifi Developmental Inventory^6^ and the Rapid Neurodevelopmental Assessment Tool.^7^ These instruments do not have proprietary restrictions and include items suitable for children in multiple settings.

Complementing these more individually focused instruments, several initiatives recently have created population-based measures of child development for children from 0 to 3 years across the world. These projects include the WHO’s indicators of Infant and Young Child Development,^8^ the Caregiver Reported Early Development Instrument (CREDI)^9^ and the Global Child Development D-Score consortium.^10^ The three groups who designed the three aforementioned studies have come together to create one tool that could be used globally—the Global Scales for Early Development (GSED) and related instruments, using existing empirical evidence from diverse settings.

### Aims and objectives

The aim of this study was to create two preliminary measures: (1) the GSED Short Form (GSED-SF)—a caregiver reported measure for population-evaluation—self-administered with no training required and (2) the GSED Long Form (GSED-LF)—a directly administered measure for programmatic evaluation—administered by a trained professional. The GSED was created using a novel, robust and reproducible methodology which combined extensive subject matter expert (SME) input with the quantitative psychometric properties of items previously chosen^11^ through a modified Rasch modelling process.^10–12^ These two measures (GSED-LF and GSED-SF) are intended to be open-access, comprise items that have stable parameter estimates, and be valid and reliable across different, geographic, cultural and language contexts.

The GSED has to discriminate between children of differing levels of development and have stable psychometric properties across countries, languages, and cultures. We also aimed to ensure that items cover relevant developmental domains (motor, language, cognitive, social-emotional, and adaptive skills), and are easy and feasible to administer in the field globally. The measures should be able to be provided both on paper or tablet and the GSED-LF includes a small selection of props, as part of the LF accompanying kit, used to better engage the child and support the child developmental assessment and child engagement. The two measures aim to be appropriate for use at a population level. The two measures (GSED SF and LF), used separately or combined depending on the level of precision needed, have been designed to also be sensitive enough to detect change after large scale programmatic interventions.

To create the GSED-SF and GSED-LF, the four specific objectives of this study are to:

- Match and group developmental items that measure identical or similar skills and behaviours across a set of existing instruments.
- Gain expert judgements on the feasibility of administering items in the field (eg, use of materials, cultural correspondence, item burden, training complexity).
- Provide information about measurement of developmental domains.
- Create prototypes of the GSED-SF and GSED-LF, using available psychometric and SME elicited information, ensuring balance across domains and a feasible selection of culturally and age-appropriate items.

Ultimately, in addition to the two stand-alone measures, we intend to incorporate the GSED-SF within the GSED-LF for programmatic evaluation to allow for even more precision having more items for a given age range. The use for programmatic evaluation of either GSED SF or LF alone should not be ruled out completely. However, we currently recommend their use together as it will provide a more precise estimate of children’s ability (given the larger number of items administered per child and added direct observation of the competencies). At this stage of measure creation, our primary objective is the assessment of stable item functioning across countries. We are therefore presently testing and validating items from the tools separately: to have to repeat the same caregiver reported SF items within the LF would be overly burdensome to participants. Furthermore, assessing items that are parent-report in the SF alongside items capturing similar or overlapping skills as direct assessment in the LF, will provide additional information for the GSED team as to which items are most valid for measuring child development and will therefore ultimately be incorporated in the revised version of GSED LF.

## Methods

### Overview

The initial psychometric modelling for the GSED measures, completed prior to this study, resulted in the construction of an ‘item bank’ that included a collection of individual items from existing ECD measures which had been shown to measured child development well, with evidence of stability across countries.^11^ The construct underpinning this item bank is a unidimensional conceptualisation of ‘early child development’ for children aged 1 month to 36 months (see van Buuren *et al*^11^: for justifications and details). The items in the item bank were selected via a modified Rasch^13^ modelling procedure that allowed the synthesis of data from 66 075 children assessed on 2211 items from 18 validated ECD measures in 32 countries. After Rasch analysis, the item bank retained 807 items from the original 2211 analysed as construct relevant measures of ECD. The next stage of the GSED development—and focus of this study—was to construct versions of the GSED-SF and GSED-LF, ensuring that the items within the scale had adequate psychometric properties and could be used to create a valid, precise and reliable measures.

**Figure 1:**
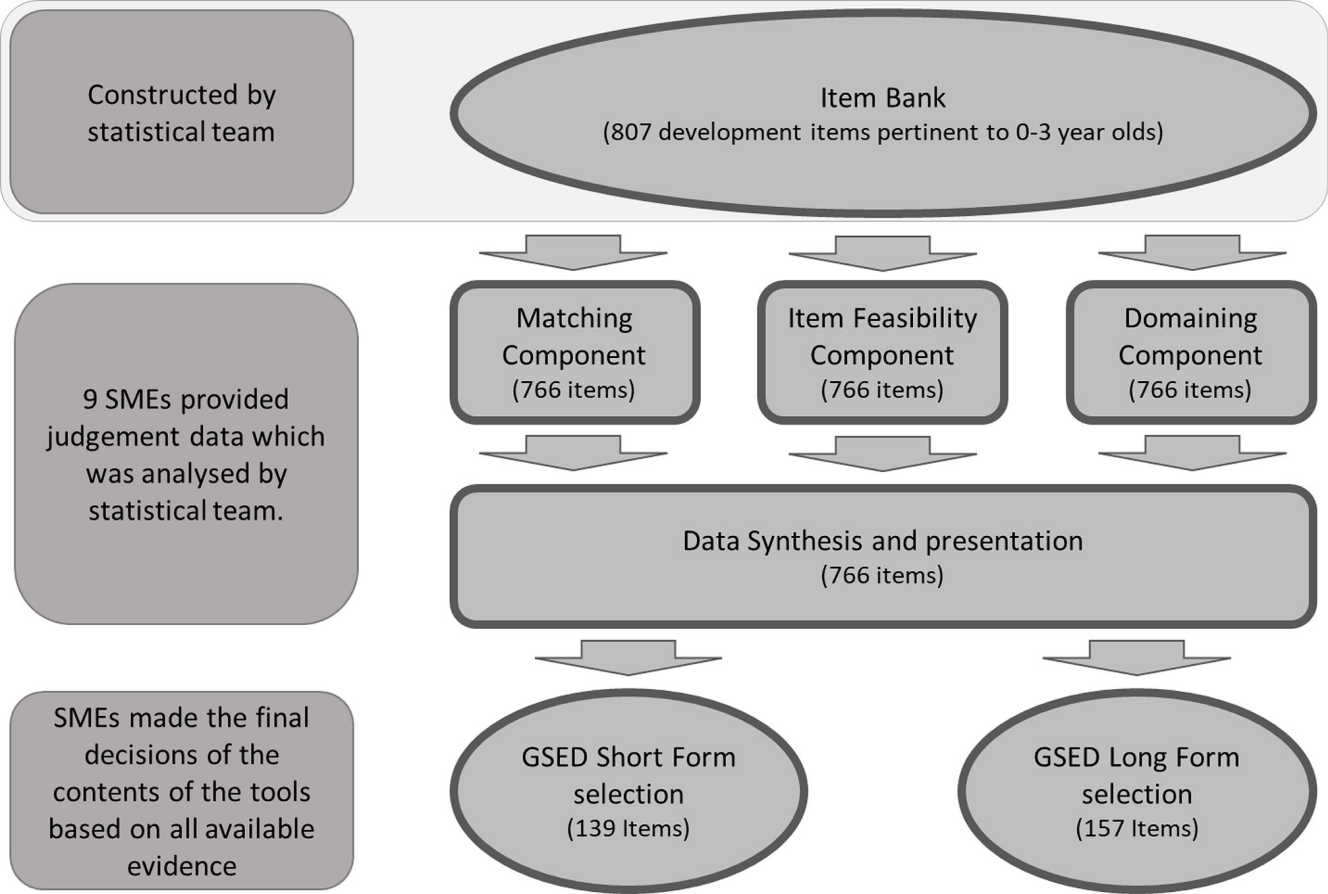
GSED Long- and Short-form creation flowcharts.

Figure 1 provides a visual outline of the methodology used to develop the GSED measures. The item selection process started with the above described item bank of 807 child development items, all of which met the criteria of a unidimensional Rasch model and were stable (based on the difficulty parameter estimate) across geographical locations, languages and cultures.^11^ Sufficiently detailed descriptors of the targeted behaviour were available for 766 of the 807 items. Nine experienced SME from relevant disciplines in early education (Developmental Psychology, Paediatrics, Economics, Epidemiology, Global Health and Education) provided judgement data on the 766 items based on three components: (1) item matching (Note: only six of the nine SMEs provided data for this component)—flagging sets of items measuring identical or similar skills or behaviours, (2) item feasibility—screening of practical considerations for implementing items globally and (3) item domain—perceptions of the developmental domain or domains each item measured. Data on these components were gathered in parallel. Psychometric information of the age-appropriateness of each item^11^ was displayed alongside the SME generated data. A web-based R Shiny Dashboard^14^ was constructed to aid in the synthesis of the judgements and item selection. Finally, SMEs used the synthesised quantitative and qualitative item information to select the items to be included in the GSED-SF and GSED-LF measures (V.1) to be field-tested in seven (The validation study is conducted in Bangladesh, Brazil, China, The Ivory Coast, The Netherlands, Pakistan and Tanzania with n=1248 children in each country) countries.^15^ The measures were created for use in a tablet-based format, accompanied by pictures and sounds to facilitate administration for GSED-SF and GSED-LF, along with materials for supporting directly administered assessment and pictures for GSED-LF. Additionally, a pack of materials for a paper-based version of the GSED was also created.

### Patient and public involvement

Patient and public involvement (PPI) was not included as part of the GSED work covered in this paper. However, extensive PPI was undertaken in the pilot phase (publication forthcoming) of the measure in order to better tailor the versions of the tool for specific countries.

### Objective (A) item matching

The item matching component was designed to establish the strength of the relationship between pairs of items across different instruments in the GSED item bank based on the skills and behaviours measured and then to group items into ‘clusters’ measuring similar behaviours. While substantial overlap in behaviours measured across the instruments, there were nuanced differences. For example, there were 58 separate items pertaining to walking, including: (1) ‘walks without falling down often’, (2) ‘walks on an uneven surface’ and (3) ‘walks well by self’. The matching exercise used a methodologically robust method^8^ of assessing the perceived degree of conceptual overlap between items measuring similar behaviours. This process was intended to facilitate the selection of a small number of items among those measuring the same general construct, preventing duplication, and thus ensuring content validity by ensuring a range of distinct behaviours.

The matching exercise was administered to SMEs via a spreadsheet that contained a matrix of all possible item pairs in the item bank; see online supplemental file 1 for an illustration and description of the components of the matching matrix and wordings of the descriptors. The designation of a match was performed according to three criteria:

10.1136/bmjgh-2022-009827.supp1 Supplementary data

1. Behaviour—the extent to which two items were measuring the same underlying behaviour, for example, walking, saying words, expressing sympathy, etc.
2. Wording—the extent to which the wording of the items led to the measurement of precisely the same underlying behaviour, for example, ‘puts hands in mouth’ is different from ‘puts hands to mouth’.
3. Facility—the extent to which both items required the same amount of support for the same quantity of a behaviour. For example, ‘walks with support’, was judged easier than ‘walks without support’. Similarly, ‘says one word’ was judged as easier than ‘says ten words’. Facility was only relevant when two items were measuring roughly the same behaviour.

SMEs rated the level of matching between every item pair in the matrix on a scale of (1) ‘very strong match’, (2) ‘strong match’, (3) ‘partial match’ and (4) ‘no match’ (see online supplemental file 1 for definitions). SMEs were instructed to mark the lowest level of match for a given item pair across the three criteria. Once collected, all pairs of matching items were extracted and the strength of the match was averaged across all judges and scaled to between 0 and 1, with 0 being ‘no match’ and 1 being ‘very strong match’. After the initial data were collected and analysed, we undertook a ‘second pass’ in which individual SMEs reviewed the collated results from all SMEs and could correct judgements perceived to be erroneous. In this second pass, all SMEs viewed all the anonymised judgements made by all other SMEs.

### Objective (B) item feasibility

The item feasibility component was designed to provide SME judgement data on the appropriateness of each item for capturing development across various geographic, cultural and language contexts. The data were used to identify for removal items that were in the item bank but were unsuitable for the diverse contexts in which the GSED instruments would be used. The SMEs considered five criteria to assess feasibility for the GSED-LF:

1. Difficulties in translation—for example, items about the definite and indefinite article (‘the’ and ‘a’/’an’) do not translate into languages that lack the definite and indefinite article.
2. Requires major adaptation to specific contexts—for example, an item that asks whether a child can use a spoon would need adapting to contexts where spoons are not commonly used.
3. Difficulties in administration/observation—for example, for a child to walk up stairs might be difficult in a flat location with few multi-story buildings.
4. Difficulties in obtaining materials—for example, items using specific materials such as a ‘plastic duck’ or a ‘screw toy’.
5. Other Concerns—any additional concerns about an item.

The GSED-SF criteria included 1, 2 and 5, from above but also added the following:

1. Caregivers may not know this about their child—for example, an item asking whether children can ‘walk sideways’ is unlikely to be known and able to be reported as a caregiver response items as it is an unusual behaviour.

Data for item feasibility were collected via online survey (see online supplemental file 2) for an example). For each item, SMEs could note any concern on a given criterion and also add a comment. These concerns were collated into an easy-to-reference document for use during the item selection procedure. Additionally, total numbers of concerns for each item were calculated and the comments were collated to be readily accessible for insertion into the data synthesis step of the methodology. Items were presented in random order in this component and in the domaining component, below, to minimise any ordering effects.

10.1136/bmjgh-2022-009827.supp2 Supplementary data

### Objective (C) item domaining

Finally, the domaining exercise was designed to provide SME judgements on the developmental domains measured by the items. Although we aimed to construct a unidimensional instrument (see van Buuren *et al*^11^ for details of the rationale and implementation of unidimensionality) with a single overall developmental score, we needed to ensure that the instrument represented all relevant developmental domains across the age range. Data were collected via online questionnaire. The taxonomy of domains was adapted from the CREDI project.^9^

There were five primary domains with several secondary domains:

1. Motor: Gross, fine.
2. Language: Receptive, expressive.
3. Cognition: Problem solving/reasoning, executive function (eg, attention, memory, inhibition), preacademic knowledge (eg, letters, numbers, colours).
4. Socioemotional: Emotional and behavioural self-regulation (eg, controlling emotions/behaviours), emotion knowledge (eg, identifying emotions), social competence (eg, getting along with others), behaviour challenges/problems—internalising (eg, withdrawal, sadness), behaviour challenges/problems—externalising (eg, hitting, kicking, biting).
5. Adaptive: Life skills (eg, using toilet, dressing).

SMEs were invited to suggest additional domains not present in the taxonomy. Items could also be ascribed to multiple domains, as many of the items measured multiple domains. For example, the item ‘Can the child tell you when others are happy, angry or sad?’ was judged as measuring both language: expressive and socioemotional: identifying emotions. Both the ability to verbally express and recognise emotions are required to pass this item. Data were collated, analysed and presented in an easy-to-access document with the numbers of ‘votes’ for each subdomain recorded. Domain representation of each item in each subdomain was rescaled between 0 ‘not representative of this domain’ and 1 ’very representative of this domain’ for further analysis by dividing the number of ‘votes’ for each item in each domain by the maximum number of votes.

### Objective (D) data synthesis and prototype creation

#### Data synthesis

The SMEs’ data were combined with the psychometric data (ie, item difficulty estimates which relate the probability of passing an item to the developmental level of the child^11^) in an R Shiny Dashboard. This online dashboard offered various functions to guide the SMEs through the final item selection process. The dashboard was written as a webapp and hosted on the Netherlands Organisation for Applied Scientific Research (TNO) private servers accessible only by the GSED team. GSED prototype creation comprised three steps:

1. Selection of a single item from a group of items for measuring a given behaviour.
2. Selection of items for a given form (ie, GSED-SF/LF).
3. Evaluation of the psychometric properties and domain coverage of the selections.

SMEs iterated over item selection and evaluation until a suitable final set of items was identified that covered the target age range and the domains within each age (see van Buuren *et al*^11^ for a full list of instruments).

##### Step 1: Best matched item selection

Two teams of constructors (from the nine SMEs) were chosen, based on their experience with the construction of similar measures, to select items for the final GSED-SF and GSED-LF. Constructors chose the most appropriate wording from items considered to be measuring the same behaviour, based on SME judgement, for the final item selection. For example, there were seven items, all from validated source instruments (see table 1 for item specifics), which measured the ability of a child to ‘copy a circle’. Having all seven flagged and presented together allowed instrument constructors to see all similar items together when deciding on the precise wording of the GSED version of the item. Items with the simplest and clearest wording were retained.

**Table 1:** Illustrative examples of items measuring ability to draw a circle

Measure	Item name	Descriptor
IYCD^21^	Copies a circle	If you draw a circle does your child do it, just as you did?
Denver^22^	Copies a circle	Give the child a pencil and piece of plain paper. Show him/her the circle on the back of the test form. Without naming it or moving your finger or pencil to show how to draw it, tell the child to draw one like the picture. Three trials may be given. 1 point: any form approximating a circle that is closed or very nearly closed. 0 points: lines that do not look close to a circle; continuous spiral motions.
ASQ3^16^	Copies a circle	Question asking the mother whether child copies a circle and providing a description of how to demonstrate it to the child.*
Griffiths^23^	Copies a circle - primitive model. Stage I.	Item asks examiner to demonstrate drawing a circle and describes options for passing and failing depending on the shape of circle.*
MDAT^5^	Copies a circle	Material: Paper, pen/pencil or chalk Instructions: Put a paper in front of child and draw a circle, explaining to the child what you are doing. ‘See how I am drawing a circle?’ You can do this up to TWICE. Then, ask child to make one just like yours. ‘Now can you draw a circle like mine?’. You can allow up to three trials for the child to make a circle. Scoring: Score YES for any nearly complete or complete circle.
KDI^6^	Can imitate a circle	No specific instructions
TEPSI^24^	Child copies a circle	‘The tester shows slide/figure 2 to the child. He gives him/her/child the pencil and the back of the record form sheet for him/her/child to draw on, and tells/asks him/her/child: ‘Draw a circle (ball, ring) like this one’

*Note: The wording of this question has been paraphrased to avoid copyright issues.

IYCD, Infant and Young Child Development; KDI, Kilifi Developmental Inventory; MDAT, Malawi Developmental Assessment Tool.

**Figure 2:**
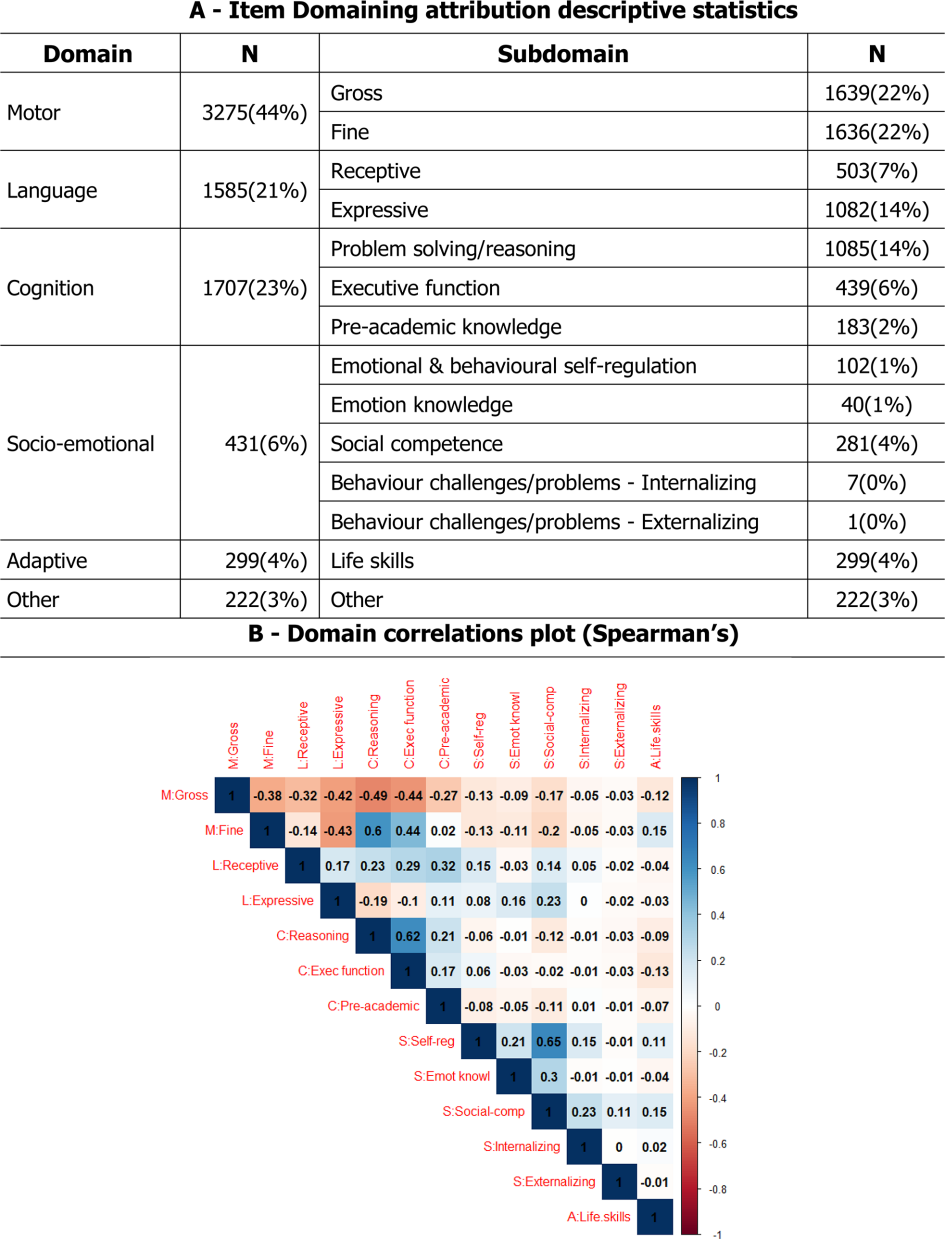
- Item Domaining attribution descriptive statistics and correlations.

##### Step 2: Item selection and allocation

Online supplemental file 3 shows the layout of the item selection page. Each item was given a study unique id, and a label summarising what it measured. Note that further information on the exact wordings of individual items was also available to the instrument creators in a different sheet of the Shiny Dashboard. The modality of the item, that is, whether the item was caregiver reported or directly administered, was displayed, followed by the domain that received most SME votes. The number of feasibility comments for each item was displayed, with the specific feasibility comments for each item available in external documentation. Finally, the psychometric information including the ages at which 10%, 50% and 90%, of participants would be expected to pass this item were given. Instrument creators selected items that could measure approximately uniformly across the entire age range while attempting to ensure appropriate domain coverage.

10.1136/bmjgh-2022-009827.supp3 Supplementary data

##### Step 3: Selected form evaluation

After a specific set of items had been selected, we conducted a set of evaluations. The evaluations included plots of the derived Development for Age Z-scores (DAZ), that is, scores on the GSED which had been adjusted for age to provide a standardised development score regardless of age,^11^ measures of reliability (separation and SE of measurement), DAZ corelations between the selected items compared with those from the model on the 807 item bank, and visualisations of the information measured by the instrument according to domain representations of the items across the age range.

Items for the first versions of GSED-SF and -LF to be used for field testing and validation were initially selected by the GSED-SF and GSED-LF constructor. The specific methods for the selection of the final items differed slightly between the forms.

#### General item selection procedures

Following steps 1–3 (above), for each form of the GSED, items that were parsimonious, had few duplications and reflected the feedback from SMEs, were chosen. The constructors reviewed the list for age coverage, conceptual integrity and domaining, ensuring that easier items preceded difficult items representing similar skills. For example, an item addressing ‘saying 5 words’ was placed earlier than ‘saying 15 words,’ regardless of the modelled difficulty score.

#### SF item selection procedure

Since GSED administration used a stop rule (ie, a rule dictating after how many contiguous items answered negatively to stop administering the GSED), constructors also chose to ‘break up’ strings of four or more items from the same developmental domain by moving down an item with a lower difficulty from another domain. Doing so ensured that a child who struggled in only one domain would not have the assessment cut short prematurely. Additionally, items that would benefit from audio-visual enhancement (eg, pictures/videos demonstrating behaviour) were flagged so that prompts could be constructed to enhance the clarity of the item.

Items in the first age range of 1–3 months had low coverage compared with the rest of the age ranges. An additional review was therefore carried out An additional review of several sources (Ages and Stages Questionnaires,^16^ CDC Milestones,^17^ Rourke Baby Record,^18^ Brazelton Neonatal Behavioural Scales^19^), resulted in seven additional items being added for this age range, for which difficulty parameter estimates were absent. The full set of items was reviewed and edited, as needed, for consistency in language across the whole measure (eg, items stating ‘the child’ were changed to say ‘your child’). Finally, preliminary conditional start and stop rules were developed. The start was set on the first item (according to our measures of item difficulty) that at least 90% of children in our existing database at the lower end of a given 6-month age bracket were able to complete. Stop was set as five contiguous ‘no’ responses.

#### LF item selection procedure

The GSED-LF complements the caregiver-reported GSED-SF and includes only items that were directly administered. In some cases, if items measuring similar areas of development were identified, but it was not clear whether an actual object or a picture of an object would be preferable, both were chosen in this prototype of the GSED-LF. For example, constructors included an item ‘identifies five objects’ and also an item ‘identifies five pictures’. The psychometric properties of both will be assessed within the validation study. Feasibility from the results of the SME survey was considered and items that were considered too culturally specific or that required complex materials administration were excluded. Items were arranged according to their difficulty level provided by the Shiny Dashboard analyses from easiest to most difficult. To ensure that (1) materials that were to be used in more than one item (eg, a cube, bean or jar) were presented parsimoniously and (2) items with similar energy levels (ie, jumping or running, vs, sitting and speaking/manipulating objects) were administered at the same time, items were partitioned into three self-similar groups.

## Results

### Objective (A) item matching results

Experts reported that the matching process took upwards of 8 hours each to complete. Three hundred and ninety-five pairs of items from the 766 items under consideration were matched and a ‘matching’ coefficient calculated. Illustrative examples of item pairs with high medium and low matching coefficients are shown in table 2. Conceptual overlap between items pairs can be seen decreasing as the coefficient moves towards zero. Ninety-eight ‘equate groups’, items which jointly had high matching coefficients and were measuring a closely related concept, were extracted by grouping clusters of items which jointly had matching levels ‘strong’ and/or ‘very strong’. Some illustrative examples of the kinds of item clusters extracted are:

1. Rolling type items:
  - Rolls from back to stomach. (Bayley3)
  - Rolls from back to stomach. (Bayley1)
  - Can roll from back to stomach, etc. (Griffiths)
  - Rolls over from back to front. (MDAT)
2. Pulling to stand items:
  - Raises self to standing position. (Bayley3)
  - Pull to stand. (Denver)
  - Pulls self while holding on to object into a standing position. (DMC)
  - Pulls self to stand/trying to get to standing. (MDAT)
3. Putting block in cup:
  - Puts cube in cup on command. (Bayley1)
  - Puts cube in and out of a box. (DDI)
  - Put block in cup. (Denver)

**Table 2:** Illustrative examples of item matches

Item 1	Item 2	Coefficient
Does your child show sympathy or look concerned? (IYCD^21^)	Does the child show sympathy or look concerned when others are hurt or sad? (CREDI^25^)	1
Does your child turn his/her head towards your voice or some noise? (IYCD^21^)	Turn to Voice (Denver^22^)	0.94
Can the child walk backwards? (CREDI^25^)	Child walks backwards unassisted>4 steps in a straight line (TEPSI^24^)	0.56
Does your child use two WORDS together in a meaningful phrase/speak in short two word sentences? (IYCD^21^)	Use a short sentence (Vineland^26^)	0.44
If you point to an object, can the child correctly use the words ‘on,’ ‘in’ or ‘under’ to describe where it is? (CREDI^25^)	Child correctly uses preposition ‘behind’ (TEPSI^24^)	0.17
Does your child try to move his/her head (or eyes) to follow an object or person? (IYCD^21^)	Eyes fixate (DDI^27^)	0.06
Does your child tell a story? (IYCD^21^)	Can the child sing a short song or repeat parts of a rhyme from memory by him/herself? (CREDI^25^)	0.00

CREDI, Caregiver Reported Early Development Instrument; IYCD, infant and young child development.

### Objective (B) item feasibility results

Table 3 lists the types and number of feasibility comments made by the SMEs. Among the directly administered items for the GSED-LF, the most commented on feasibility criterion was the ability to obtain the required materials and for the caregiver-reported SF measure it was the category ‘other concerns’, which comprised uncategorised comments. The directly administered item with the most feasibility comments (9 comments made), was ‘Points to Five Pictures’. The comments mainly concerned the difficulty in selecting images that would allow this item to be cross-culturally comparable. The caregiver-reported item with the most feasibility comments was related to transferring objects. Most of the feasibility comments related to the ambiguousness of what was being asked and the complexity of language in the scoring rubric.

**Table 3:** Types and examples of comments for each feasibility criterion

Direct administration(n=615)					
	Difficulties in translation	Requires major adaptation	Difficulties in administration	Difficulties in obtaining materials	Other Concerns
No of comments	14 (1%)	230 (19%)	180 (15%)	420 (34%)	396 (23%)

	Caregiver report (n=151)			
	Difficulties in translation	Requires major adaptation	Caregivers may not know this	Other Concerns
No of comments	27 (10%)	55 (20%)	66 (24%)	129 (47%)

Example comments		
	Item	Comment
**Direct administration**		
Difficulties in translation	Refers to self, using 'me' or 'I' (can ask parents)	‘In some languages, pronouns are not used, because the person is indicated by the form of the verb.’
Requires major adaptation to context	wave bye-bye	‘Might need to be changed slightly if waving not common in all cultures.’
Difficulties in administration.	Moves from lying to sitting pushing up with hands.	‘Difficult to evoke this behaviour on command.’
Difficulties in obtaining materials.	Look for yarn.	‘Would want to replace yarn with something else.’
Other concerns	Imitates a two-word utterance.	‘May be hard to administer to very shy children.’
**Caregiver report**		
Difficulties in translation	makes sentences that are three or four words long.	‘May need to be adapted in contexts that include verb conjugation that would span 3–4 words in English.’
Requires major adaptation to context	When you ask, ‘What is your name?’ does your child say both her first and last names?	‘Some cultures use multiple names, so we would need to specify which names would be acceptable responses.’
Caregivers not know this about their child.	Can the child unscrew the lid from a bottle or jar?	‘May not have observed or identified this specific behaviour. Might be better as direct assessment.’
Other Concerns	Does your child put objects or hands to his/her mouth?	‘Had problems with this question in certain settings - mothers said ‘no’ because they thought that it was unhygienic.’

Examples of comments made for each feasibility criterion and each modality are given in table 3. The feasibility criteria elicited useful information from the experienced SMEs. The feasibility results were tabulated, and a reference document was constructed with all feasibility comments for each item.

### Objective (C) item domaining results

Domaining results were extracted, and a domain profile was constructed for each item indicating the SMEs’ assessment of developmental domain. Figure 2 Panel A gives the numbers of individual votes for items broken down by both domain and subdomain. Items that the SMEs judged as measuring motor skills made up almost half of the item bank, followed by cognition and language. Looking at subcategories, some of the socioemotional subdomains had little representation in the item bank so we analysed at the domain level. The balance of items across domain was not uniform across age. As expected, given normative developmental progressions, at younger ages there were more motor items and at older ages, there were more language and cognitive items.

To investigate which domains were jointly ascribed to a single item with a higher frequency, a correlation matrix was computed at the subdomain level. Spearman’s correlations between the domain ascriptions are shown in figure 2 Panel B. Gross motor negatively correlated with all other subdomains indicating that when an item measures gross motor, it tends to measure only gross motor. In contrast, fine motor correlated positively with reasoning, executive function, and life skills-tasks, indicating that items measuring fine motor often also measure the correlated sub-domains. For example, the item ‘Can manage a cup well’ was judged to measure both fine motor and life skills.

### Objective (D) data synthesis and prototype creation results

#### GSED short form

The resulting GSED-SF contains 139 caregiver-reported items using a yes/no response scale. All items (Note: for seven items for younger children, we had no domain information as they were added after the domaining study had been completed) reflect basic skills, behaviours and milestones that caregivers are likely to know and be able to report about their children. Items collectively cover all domains of development. Many (54/139; 39%) have accompanying audio or visual clues that are presented to the caregiver while they are being interviewed and are easily incorporated into an online version of self-report.

#### GSED long form

The resulting GSED-LF consists of 157 directly administered items in three groups that were chosen to facilitate flow of administration (49 items in group A, 54 items in group B and 54 items in group C). Group A mostly includes items requiring physical activity or movement (motor items), Group B mostly includes items where the examiner needs to listen or speak with the child or interact using the tablet format (language items), and group C mostly includes items where the examiner uses materials from the kit with the child. A first version of the kit for the GSED was created to accompany the GSED-LF. The kit includes: a timer, a measure of 2 m in string, a ball, a rattle, some small 2 cm square blocks, a spoon, a plate, a cup, a crayon/pencil, pegboard, shape-board and items for naming for example, comb, car, cloth, shoe, bottle—items were selected to be freely available in all sites.

## Discussion

We have created two measures of early child development for children between 0 and 3 years (0–36 months) for use at the population and programmatic level through a data-driven approach. A large dataset was used alongside SME judgements to select items with strong psychometric properties across various geographic, cultural, and language contexts. The original GSED dataset used to identify developmental items contained 66 075 children assessed on 2211 items from 18 ECD measures in 32 countries.^11^ The analytical process combining multiple instruments onto a common scale, as well as using a robust process for collecting detailed SME judgement information to facilitate item selection is, to date, the most ambitious and far-reaching data-driven test construction process the field has seen. Using this approach, we have created measures with items that have excellent feasibility and comprehensively represent age-appropriate content for the measurement of child development demonstrated to be stable across various contexts. This methodology, which depended on SME expertise and effort, allowed for transparency and reproducibility. The extensive empirical and qualitative information available about each item provides a unique opportunity to create valid, reliable and cross-culturally feasible measures of ECD.

In contrast with many other measure construction studies, we utilised an individually elicited judgement gathering processes. This anonymised elicitation process reduced potential interference from the opinions of other SMEs in the initial phase. Furthermore, we created a process of randomisation of the presentation of items in the feasibility and domaining components that reduced systematic bias regarding the information collected on the items. The judgement data provided by the SMEs is a valuable resource for test constructors, researchers and academics to use in the future.

Unsurprisingly, in the assessment of children aged 0–3 years, many of the developmental items in the GSED cross-over domain boundaries. Brain development proceeds along species-specific pathways with acceleration in certain areas mirrored by the skills that children acquire and demonstrate at specific ages. For example, motor areas of the brain show earlier myelination than temporal and frontal areas that are necessary for language and cognition.^20^ The balance of items in GSED-SF and GSED-LF mirrors this pattern, with a wide range of motor items in the earliest months, followed by language and cognitive items in the later months. Reflecting caregivers’ knowledge about children’s social and relational skills, socioemotional and adaptive items were predominantly included in the GSED-SF rather than in the GSED-LF.

A limitation of this procedure for tool creation is that it requires a vast amount of resources to implement, both in terms of SME time and the gathering and linking of the items for the dataset that underpinned it. Furthermore, in order to validate these tools globally, a great deal of work must be done to collect and analyse item response data from many countries to check the consistency of the performance of the tools. Creating a tool in the manner, we have described requires significant funding, expertise, and commitment by study participants for which we are truly grateful.

In conclusion, through robust methodological processes, including both quantitative and qualitative information, we have selected items to construct a set of measures of ECD for global use covering the age range of 0–3 years. These measures represent theoretical domains underlying early development, measured by items that are feasible for administration in different global contexts. The versions of the GSED-SF and GSED-LF created by the WHO through these processes will be piloted and validated in children across multiple countries, both lower income and middle income and higher income, to ensure validity across diverse demographic, social and language contexts for future use.

## Data Availability

Data are available on reasonable request.
