# Supplementary material for: The creation of the Global Scales for Early Development (GSED) for children aged 0–3 years: combining subject matter expert judgements with big data
Source: BMJ Glob Health. 2023 Jan 17;8(1):e009827. doi: 10.1136/bmjgh-2022-009827 (PMC9853147; doi:10.1136/bmjgh-2022-009827)
Supplement: Supplementary data [file bmjgh-2022-009827supp001.pdf]

[redacted]

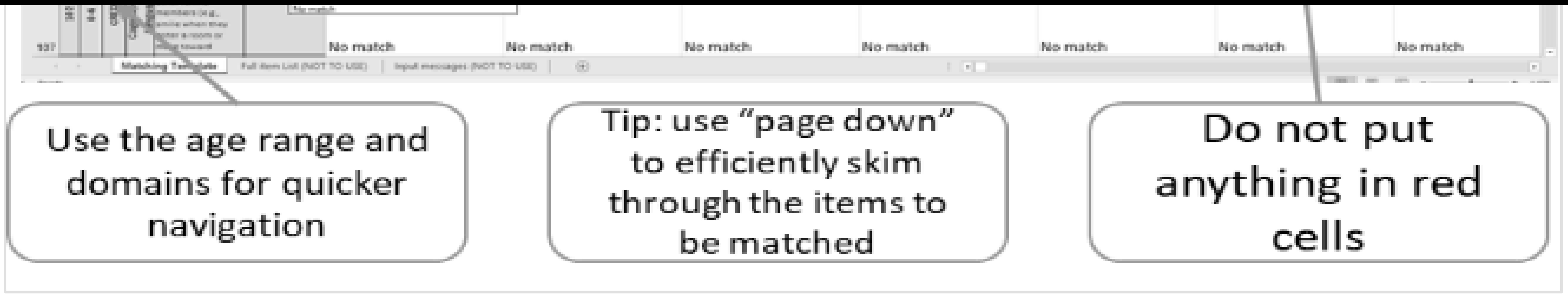

Criteria	Very Strong	Strong	Partial	No match
**Behaviour**	Measures the same underlying behaviour	Measures a similar underlying behaviour	Measures a somewhat similar underlying behaviour	Does not measure the same behaviour
**Wording**	Very few or no words difference	Some words difference	Many words difference	Very different wording
**Facility**	Measures exactly the same level of facility (if applicable)	Measures a similar quantity level of facility (if applicable)	Measures a somewhat different level of facility (if applicable)	Measures a very different level of facility (if applicable)
