# Supplementary material for: The creation of the Global Scales for Early Development (GSED) for children aged 0–3 years: combining subject matter expert judgements with big data
Source: BMJ Glob Health. 2023 Jan 17;8(1):e009827. doi: 10.1136/bmjgh-2022-009827 (PMC9853147; doi:10.1136/bmjgh-2022-009827)
Supplement: Supplementary data [file bmjgh-2022-009827supp002.pdf]

GSED-LF

Question
"Can the child count up to five objects (e.g., fingers, people)?"
Modality: Caregiver Report, **Response options:** Yes/No

Feasibility

	Level of Concern		Rationale for concern
	Not Concerned	Concerned	Optional text
Difficulties in translation to certain language(s)	●	○	
Requires major adaptation to specific contexts (e.g., not culturally appropriate)	●	○	
Caregivers will likely not know this about their child	●	○	
Other Concern [Please Specify]	●	○	

GSED-SF

Question
"Laughs aloud"
Examiner's action: Listen for the child to laugh aloud. If this is not heard, ask the caregiver if the child does his. 1 point: Hear the child laugh aloud or if the caregiver reports he/she does this. 0 points: Child is not observed laughing out loud and caregiver reports he/she does not do this.
Child's action: 1 point: Hear the child laugh aloud or if the caregiver reports he/she does this. 0 points: Child is not observed laughing out loud and caregiver reports he/she does not do this.
Modality: Direct Assessment, **Response options:** Yes/No

Feasibility

	Level of Concern		Rationale for concern
	Not Concerned	Concerned	Optional text
Difficulties in translation to certain language(s)	●	○	
Requires major adaptation to specific contexts (e.g., not culturally appropriate)	●	○	
Difficulties in the administration of item/observation of skill or behaviour	●	○	
Difficulties in obtaining/making required materials in certain context	●	○	
Other Concern [Please Specify]	●	○	
