# Supplementary material for: The creation of the Global Scales for Early Development (GSED) for children aged 0–3 years: combining subject matter expert judgements with big data
Source: BMJ Glob Health. 2023 Jan 17;8(1):e009827. doi: 10.1136/bmjgh-2022-009827 (PMC9853147; doi:10.1136/bmjgh-2022-009827)
Supplement: Supplementary data [file bmjgh-2022-009827supp003.pdf]

Show 10 entries

Search:

	item	label	modality	voted_domain	feasibility comments	tau	A10	A50	A90
320	denfmd006	Regard raisin	Direct	cog	7	32.75	3.77	4.92	6.34
428	griehd011	Hands explore table surface	Direct	motor	4	34.08	4.09	5.31	6.82
742	sgrfmd016	Grasps box.	Direct	motor	4	34.89	4.29	5.55	7.12
405	dmcsld010	Watches others and plays next to them.	Direct	sem	3	30.63	3.3	4.36	5.64
322	denfmd008	Look for Yarn	Direct	cog	3	31.18	3.42	4.5	5.82
317	denfmd003	Hold Rattle	Direct	motor	3	31.33	3.45	4.54	5.86
753	sgrred018	Manipulates cup or spoon in play	Direct	motor	3	33.56	3.96	5.16	6.62
319	denfmd005	Follow 180 Degrees	Direct	motor	3	34.5	4.19	5.43	6.97
[illegible]	[illegible]	Secures [illegible]	[illegible]	[illegible]	[illegible]	[illegible]	[illegible]	[illegible]	[illegible]
